# Hip reconstruction using a customized intercalary prosthesis with the rhino horn-designed uncemented stem for ultrashort proximal femur segments following tumor resection: a combined biomechanical and clinical study

**DOI:** 10.1186/s12891-022-05805-9

**Published:** 2022-09-08

**Authors:** Xin Hu, Minxun Lu, Xuanhong He, Longqing Li, Jingqi Lin, Yong Zhou, Yi Luo, Li Min, Chongqi Tu

**Affiliations:** 1 Department of Orthopedics, Orthopedic Research Institute, West China Hospital, Sichuan University, No. 37 Guo Xue Xiang, Chengdu, 610041 Sichuan People’s Republic of China; 2 Department of Model Worker and Innovative Craftsman, West China Hospital, Sichuan University, No. 37 Guo Xue Xiang, Chengdu, 610041 Sichuan People’s Republic of China

**Keywords:** Intercalary endoprosthesis, Ultrashort femur segments, 3D-printed prosthesis, Finite element analysis, Bone tumor

## Abstract

**Background:**

Hip-preserved reconstruction for patients with ultrashort proximal femur segments following extensive femoral diaphyseal tumor resection is a formidable undertaking. A customized intercalary prosthesis with a rhino horn-designed uncemented stem was developed for the reconstruction of these extensive skeletal defects.

**Methods:**

This study was designed to analyze and compare the differences in the biomechanical behavior between the normal femur and the femur with diaphyseal defects reconstructed by an intercalary prosthesis with different stems. The biomechanical behavior under physiological loading conditions is analyzed using the healthy femur as the reference. Five three-dimensional finite element models (healthy, customized intercalary prosthesis with four different stems implemented, respectively) were developed, together with a clinical follow-up of 12 patients who underwent intercalary femoral replacement.

**Results:**

The biomechanical results showed that normal-like stress and displacement distribution patterns were observed in the remaining proximal femur segments after reconstructions with the rhino horn-designed uncemented stems, compared with the straight stem. Stem A showed better biomechanical performance, whereas the fixation system with Stem B was relatively unstable. The clinical results were consistent with the FEA results. After a mean follow-up period of 32.33 ± 9.12 months, osteointegration and satisfactory clinical outcomes were observed in all patients. Aseptic loosening (asymptomatic) occurred in one patient reconstructed by Stem B; there were no other postoperative complications in the remaining 11 patients.

**Conclusion:**

The rhino horn-designed uncemented stem is outstanding in precise shape matching and osseointegration. This novel prosthesis design may be beneficial in decreasing the risk of mechanical failure and aseptic loosening, especially when Stem A is used. Therefore, the customized intercalary prosthesis with this rhino horn-designed uncemented stem might be a reasonable alternative for the reconstruction of SSPF following extensive tumor resection.

## Introduction

Hip-preserved reconstruction for the extremely short bone segments in the proximal femur (SSPF) caused by extensive tumor resection of the diaphysis is technically demanding. In our institution, we defined an SSPF as the length of the remaining proximal femur (from the pyriform fossa to the osteotomy level) of ≤ 80 mm. Currently, there are two types of solutions would be available for the reconstruction of SSPF. The biological reconstruction with allograft or autograft is a considerable approach, but the relatively higher risk of infection and nonunion are unacceptable. While a prosthetic replacement has become the mainstream. In total, there are four choices of the prosthesis in this method, including the custom-made/modular megaprosthesis [1], allograft prosthetic composite (APC) [2], Compliant Pre-stress (CPS) implant [3], and the intercalary prosthesis [4–7].

Megaprosthesis replacement is a regular modality for the reconstruction of tumorous massive defect of the femur. This reconstruction method requires a sufficiently long length of the remaining bone (≥ 10 mm) to be fixed with the femoral stem to achieve stable fixation [8]. In a study investigating the relationship between the length of the femoral stem and aseptic loosening, Zhang et al. reported that every 1 mm decrease in the length of the stem indicated the risk of aseptic loosening could be increased by 6% [9]. Batta et al. [10] noted that the ratio of the total length of the prosthesis to stem length (TLP/SL ratio) was an independent predictor of aseptic loosening, and as the TLP/SL ratio increases, the risk of aseptic loosening increases. Therefore, when reconstructing the SSPF with the megaprosthesis become necessary, a modular short femoral stem is currently the only option because the SSPF cannot provide enough space for a long femoral stem fixation. To address the problem of insufficient length of the remaining bone in prosthesis fixation, APC reconstruction is supposed to be an alternative method, which bridged a segment of allograft bone to the remaining femoral segments, thereby allowing the use of a distal femur megaprosthesis with a longer stem [11]. Nevertheless, APC reconstruction is vulnerable to allograft-related complications (dislocation, infection, host-graft nonunion, and fracture), restricting its clinical application [12]. The CPS implants can reconstruct an ultrashort femur segment (4–8 cm) by using a unique compressive fixation method, which can provide immediate endoprosthetic anchorage and ongoing compliant fixation [3]. It doesn’t require allograft bone grafting, thus sparing patients from these allograft-related complications. Unfortunately, 50% of patients required revision surgery within a decade after initial surgery due to spindle failure or rotational failure, limiting its clinical application [13, 14]. Overall, all the methods mentioned above necessitate the removal of the knee joint, which creates the possibility of more complications and wear debris at articulation, making the rehabilitation more rigorous and complicated.

In 1989, Chin et al. [15] first reported the intercalary prosthetic replacement and stated that this method has the advantage of restoring limb function immediately, allowing shorter hospital stays, and early rehabilitation. Most importantly, both hip and knee joints could be preserved by using this technique. However, a high rate of aseptic loosening caused by the insufficient length of the remaining bone in prosthesis fixation is also expected in the intercalary prosthetic replacement. For this reason, we developed a set of novel customized stems with different radius of curvature (ROC), namely the rhino horn-designed uncemented stems. These prosthetic stems can not only spread into the deeper region of the femoral head but can also closely match the curvature of the SSPF, thereby increasing the length of the stem and achieving stable fixation. The purpose of the present study was to evaluate the biomechanical stability and the clinical efficacy of the SSPF reconstructed by the customized intercalary prosthesis with the rhino horn-designed uncemented stem. To elucidate the biomechanics of this novel prosthetic stem and verify the influence on the biomechanical behavior of different stem ROC, we analyzed and compared differences in the biomechanical properties between the healthy femur model and four intercalary prosthesis reconstruction models through finite-element methods (FEM) [16, 17]. Moreover, a clinical retrospective study was also conducted to validate its clinical efficacy.

## Methods

### Biomechanical study

#### The novel rhino-horn designed uncemented stem

In our study, different stem ROC corresponds to different locations of stem tip where the stem was inserted in the femoral head. The underlying biomechanical mechanisms regarding different stem ROC remain unknown. Therefore, three sets of endoprostheses having different stem ROC and one standard straight stem were created, namely Stem A, Stem B, Stem C, and stem D. It would be beneficial to understand the mechanisms of this newly designed prosthesis by contrasting the mechanical performance of these three representative curved stems and one straight stem.

**Fig. 1:**
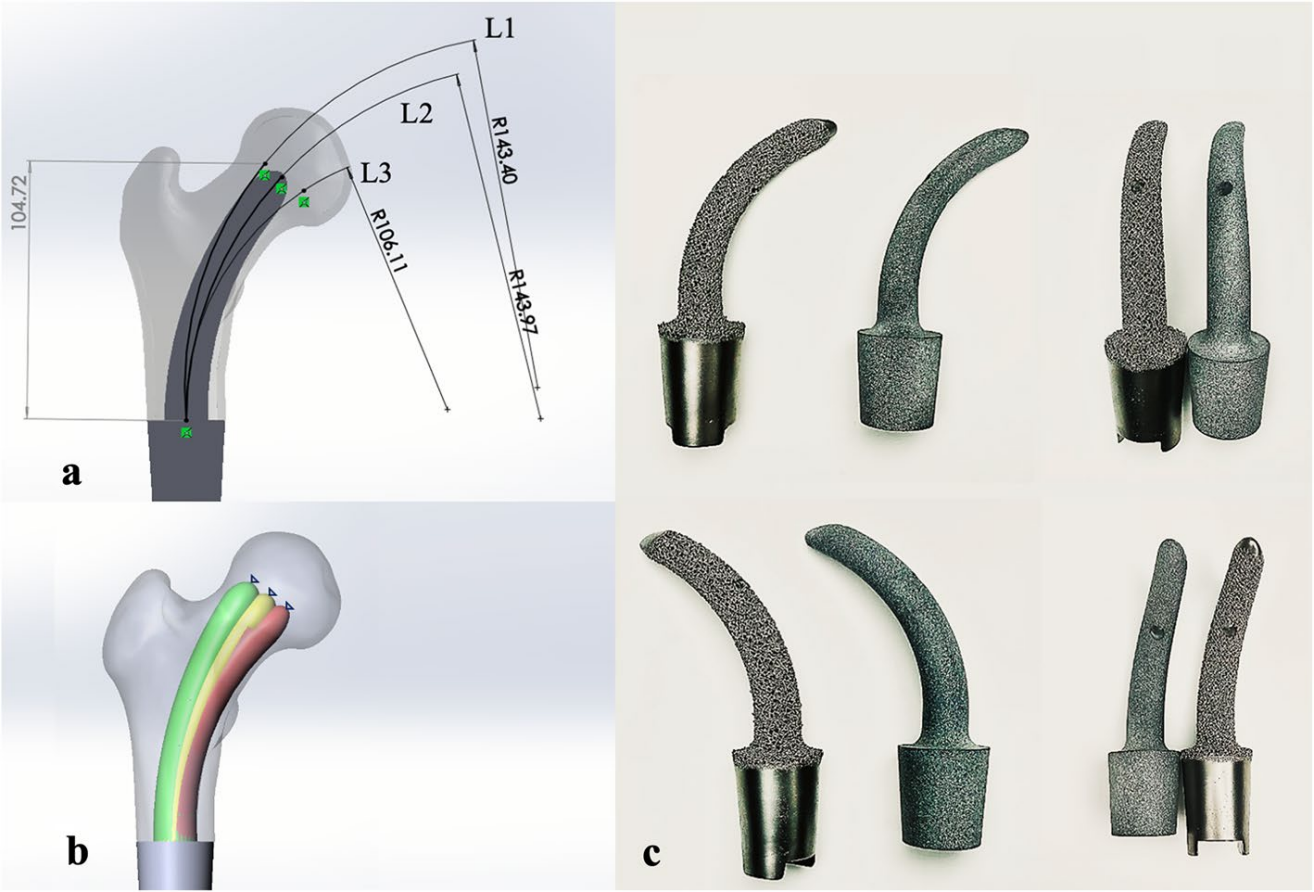
Diagram of the rhino horn-designed uncemented stem: **a** Schematic drawing of the prosthetic stem. The curved lines L1-3 represented different stem ROC, and three representative prosthetic stems were created based on those curved lines. **b** The stem tip position varied with the stem ROC; the green stem, yellow stem, and red stem represents Stem C, Stem B, and Stem A, respectively. **c** The photographs of the prosthetic stem and its plastic models

Relative to the point of the center of the femoral head, two points were set above and below the center point, at 1 cm intervals. Based on those points and the osteotomy plane, three imaginary curved lines were drawn to determine the stem ROC (Fig. 1a-b).

- Stem A was developed based on an imaginary curved line (L3) through the inferior point and the center of the osteotomy plane.
- Stem B was developed based on a curved line (L2) through the femoral head center and the center of the osteotomy plane.
- Stem C was developed based on another curved line (L1) through the superior point and the center of the osteotomy plane.

The root of the stem was designed as a cylindrical structure with a diameter close to the inner diameter of the medullary cavity at the osteotomy plane; the diameter of the stem gradually reduces from base to tip but not less than 10 mm. The diameter of the whole stem was approximately two-thirds of the diameter of the medullary cavity in the intertrochanteric region. Additionally, the stem was made up of two types of titanium-based materials, solid titanium and porous titanium [18]. The center of the stem was a solid titanium structure that can provide enough mechanical strength, while the outer layer of the stem was a porous titanium structure with a thickness of 2-3 mm, which can offer weaker mechanical strength but good osteointegration ability:

- The inner part of the stem is made of solid titanium to ensure prosthetic durability.
- The lateral part of the stem is made of porous titanium with 500 μm-pore-size and 70% porosity to facilitate osseointegration [19].
- The medial part of the stem is made of porous titanium with 400 μm-pore-size and 50% porosity to provide enough mechanical strength [19].

#### Establishment of the three-dimensional finite element models

**Fig. 2:**
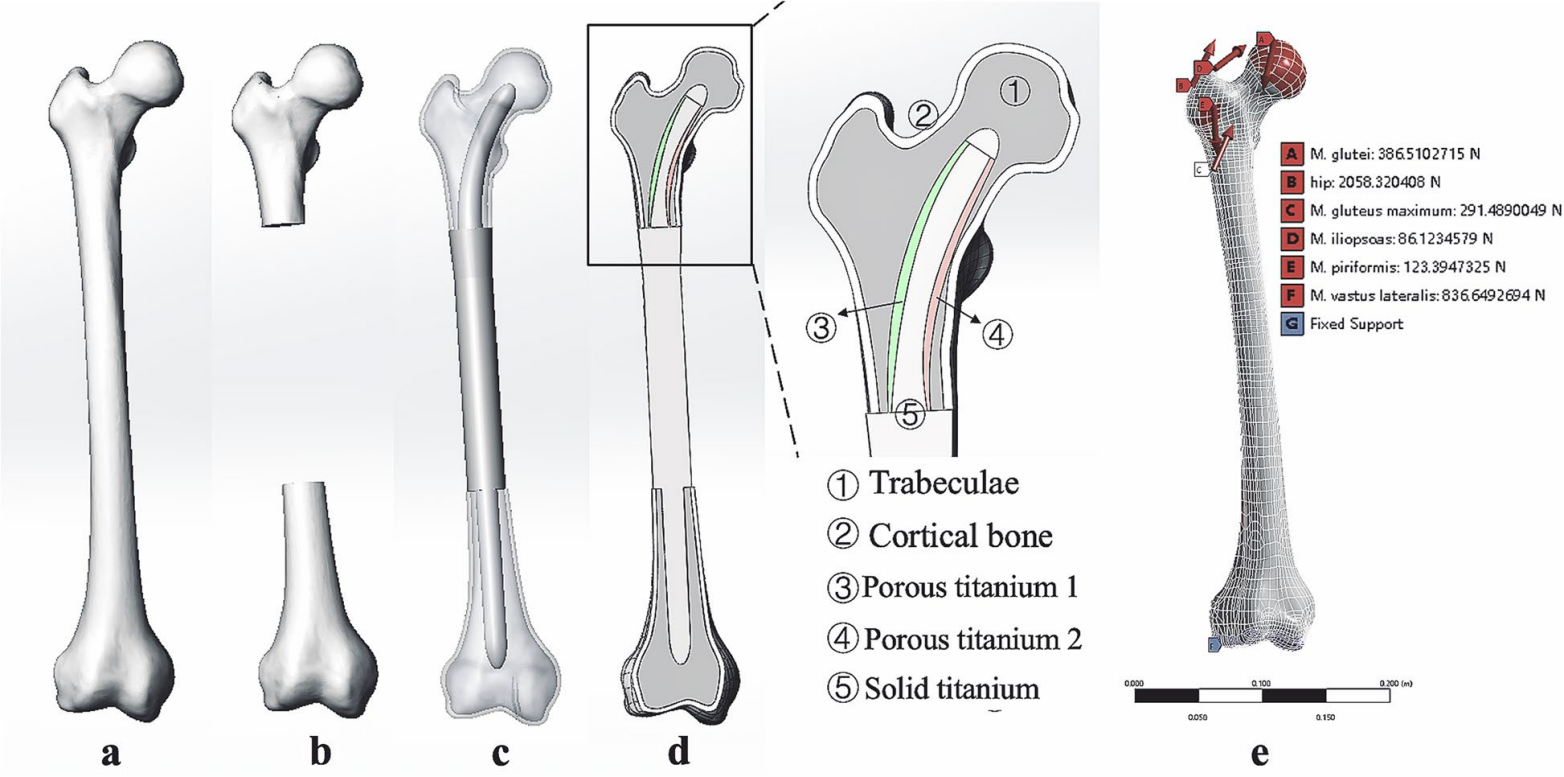
Diagram of the finite element models: **a** The normal femur. **b** The femur remaining after tumor resection. **c** The intercalary femoral replacement model. **d** Cross-sectional drawing of the intercalary femoral replacement model. The porous titanium 1 has a pore size of 400um and 50% porosity; the porous titanium 2 has a pore size of 500 μm and 70% porosity. **e** The hip joint-femur muscle multiple force was applied to the femur model while the distal condyle articular surface was fixed

The femoral CT data used in this study were obtained from a healthy adult volunteer (male, height = 172 cm, weight = 71 kg). Firstly, we imported the CT data of the healthy volunteer into Mimics V17.0 (Materialise Corp. Belgium) to create the initial normal femur model, and then it was imported into Geomagic Studio 2014 (3D Systems, Inc. USA) to optimize the model quality and complete the final model development (Fig. 2a). After the normal femur model was created, it was consequently imported into Solidworks 2016 (Dassault Systèmes Solidworks Corporation, France) to simulate the osteotomy. Since 40% of the length of the femur is the most common range of osteotomy in distal femoral replacement [20], the present study simulated the femoral osteotomy at this representative level (Fig. 2b). We created the distal part of the prosthesis based on the guidance of the manufacturers’ specifications.

The aforementioned components including the prosthetic stems, the distal part of the modular endoprosthesis, and the femur remaining after tumor resection were imported into Solidworks 2016 to assemble the intercalary femoral replacement models. All these virtual surgical procedures were guided by the surgeon (Chongqi Tu) who completed the actual surgery. Finally, five different finite element models in total were created (Fig. 2c):

- Model #O: normal femur
- Model #A: intercalary tumor resected + Stem A + intercalary endoprosthesis
- Model #B: intercalary tumor resected + Stem B + intercalary endoprosthesis
- Model #C: intercalary tumor resected + Stem C + intercalary endoprosthesis
- Model #D: intercalary tumor resected + stem D + intercalary endoprosthesis

#### Material assignment and mesh

All components were assigned isotropic and homogeneous material properties based on previously reported data [21] in Ansys 2019 R3 software (ANSYS, Inc. Pennsylvania, USA). The elastic modulus and Poisson’s ratio were set to 13.7 GPa and 0.3 for cortical bone, and 1.85 Gpa and 0.3 for trabeculae bone, respectively. Except for the prosthetic stem, the material of all modular endoprosthesis systems was titanium (Ti–6Al–4 V) with the elastic modulus = 110 Gpa, and Poisson’s ratio = 0.3. The inner part of the prosthetic stem was made of solid titanium with the elastic modulus = 110 Gpa, and Poisson’s ratio = 0.3. The medial part of the prosthetic stem was made of porous titanium with the elastic modulus = 3.5 Gpa, and Poisson’s ratio = 0.3, the lateral part of the stem was also made of porous titanium but with higher pore-size and porosity, which results in an elastic modulus of 1.12 Gpa, and a Poisson’s ratio of 0.44 (Fig. 2d and Table 1). All finite element models were meshed in ANSYS Meshing with tetrahedron elements (C3D4), and the number of elements was 489,094 for the normal femur, 594,559 for Model# A, 595,574 for Model# B, 548,106 for Model# C, and 489,094 for Model# D.

**Table 1:** material properties of the bone and implants

Materials	Modulus of elasticity (Gpa)	Poisson’s ratio
Cortical bone	13.7	0.3
Trabeculae bone	1.85	0.3
Solid titanium (Ti–6Al–4 V)	110	0.3
Porous titanium (medial)	3.5	0.3
Porous titanium (lateral)	1.12	0.44

#### Loads and constraints

The biomechanical performance of these models under physiological loading conditions was investigated. The loading conditions representing an instant at 30% of the gait cycle were implemented on the femur according to the study of Bitsakos et al. [22]. The hip joint-femur muscle multiple forces were applied according to the reported literature [23]. The direction and magnitude of the hip reaction and muscle forces are displayed in Table 2 and Fig. 2e. Additionally, the articular surface of the femoral condyle of each model was restricted.

**Table 2:** The direction and magnitude of the hip reaction and muscle forces

Muscle group/contact force (N)	Force applied (X)	Force applied (Y)	Force applied (Z)
Hip reaction force	-790	230.1	-1886.7
Gluteus maximum	169.6	-78.2	223.8
Glutei	225.7	-32.3	312.1
Iliopsoas	-0.5	65.6	55.8
Piriformis	104	-56.5	34.9
Vastus lateralis	42	-193	-813

For all finite element models, the cortical bone-trabeculae bone interface was assigned as bonded in Ansys 2019 R3, and the interface between the bone and the prosthesis was set to frictional type with a friction coefficient of 0.3.

#### Finite element solution

All models were imported into Ansys 2019 R3 finite element analysis. We identified the mechanical properties of the novel stem design with different ROC by observing the stress and displacement distribution.

### Clinical study

#### Patient demographics

Between October 2015 and 2020 December, 12 consecutive patients from our institution in which the rhino horn-designed uncemented stem was used for intercalary femoral replacement were retrospectively reviewed. Patients with incomplete follow-up data, patients with serious osteoporosis, patients with deformities of the lower limbs, and patients with metal implant allergy were excluded.

The cohort included 6 males and 6 females, with a mean age of 25.92 ± 18.50 years (range,11–77). Preoperatively, all patients underwent a complete preoperative examination, including X-ray, 3D-CT, MRI, and computed tomography (SPECT). All patients were diagnosed with primary sarcoma via biopsy prior to definitive surgery. Of these patients, 5 patients (41.7%) were diagnosed with osteosarcoma, 2 (16.7%) with parosteal osteosarcoma, 3 (25.0%) with Ewing sarcoma, 1 (8.3%) with myofibroblastic sarcoma. The tumor stages were classified based on the Enneking system. The characteristics of the patients are summarized in Table 3.

**Table 3:** Basic patient information

Patients	Sex/Age	Diagnosis	Enneking stage	Follow-up (month)	Osteotomy length (mm)	Proximal femur length after tumor resection (mm)	Full femur length (mm)	Percentage of sectional femur length in the full femur length (%)	Neck-shaft angle (°)	Anteversion angle (°)	Stem type
1	M/45	Parosteal osteosarcoma	IIB	48	185.0	72.5	425.2	43.5	124	13.2	Stem B
2	M/11	Ewing sarcoma	IIB	27	189.5	50.5	365.2	51.9	125	14.1	Stem B
3	M/30	Myofibroblastic sarcoma	IIB	24	228.4	74.4	401.8	56.8	128	12.9	Stem B
4	F/18	Parosteal osteosarcoma	IIB	31	113.8	53.0	405.2	28.1	124	14.2	Stem C
5	F/22	Osteosarcoma	IIB	30	258.4	75.8	411.2	62.8	129	14.5	Stem C
6	F/13	Osteosarcoma	IIB	48	137.5	76.5	395.7	34.7	130	13.5	Stem A
7	M/77	Liposarcomas	IIB	18	211.5	74.8	440.2	48.0	127	12.5	Stem A
8	F/21	Osteosarcoma	IIB	31	176.0	59.2	420.1	41.9	119	13.7	Stem A
9	M/18	Osteosarcoma	IIB	27	198.0	74.0	410.7	48.2	122	14.1	Stem A
10	F/25	Osteosarcoma	IIB	36	110.5	71.0	415.4	26.6	125	13.5	Stem B
11	M/15	Ewing sarcoma	IIB	28	105.0	67.0	395.2	26.6	134	14.2	Stem C
12	F/16	Ewing sarcoma	IIB	40	139.5	65.5	392.8	35.5	126	15.2	Stem C

(Proximal femur length after tumor resection = the length from the pyriform fossa to the osteotomy level)

This study was performed in accordance with the 1964 Helsinki Declaration and was approved by the Ethics Committee of West China Hospital. Written informed consent was obtained from adults or parents of minor participants (below 16 years of age).

#### Prosthesis fabrication and selection

All prostheses were customized for each patient by our clinical team and fabricated by Beijing Chunlizhengda Medical Instruments Co., Ltd (Tongzhou, Beijing, China) using the electron beam melting technique (ARCAM Q10plus; Mol̈ndal, Sweden). (Fig. 1c).

In addition, inserting Stem B into the center of the femoral head is less invasive and less technically demanding than inserting Stem A or C into the inferior or superior region of the femoral head. Therefore, the type of the rhino horn-designed uncemented stem (Stem A, B, or C) was determined by the surgeon based on patient specific factors including age, the endurance of surgery, and bone quality. For example, Stem B would be recommended for an older patient with poor cardiopulmonary function or bone quality. Otherwise, Stem A or C would be recommended.

#### Surgical techniques

All operations were performed by a senior surgeon (Chongqi Tu) from our clinic team. A lateral approach to the hip was used with a long incision on the thigh. Tumor resections were in accordance with the standard oncological principles. After the osteotomy was completed, a Kirschner wire was pre-inserted into the center of the femoral head as the channel of stem implantation. Then we used flexible reamers with different diameters to enlarge the medullary cavity of the femur, maintaining the anatomical curvature on the guidance of the Kirschner wire. After the stem was inserted into the remaining femur segments, the intercalary femoral replacement was performed by using the custom modular endoprosthesis with a suitable length.

#### Postoperative management

Prophylactic intravenous (IV) antibiotics (1,500 mg IV cefazolin sodium) were given 30 min before catheterization, then they were continued for 72 h postoperatively. Subcutaneous injections of low molecular weight heparin (2,500 IU/day) were given postoperatively until the patient was able to move without restriction.

The postoperative rehabilitation protocol was designed individually and based on the surgeon’s intraoperative assessment. After surgery, patients were required to stay in bed for 2–3 weeks with the lower extremity being maintained in a neutral position. During this period, patients were encouraged to perform flexion and extension function exercises of the knee and ankle. The partial weight-bearing stance with walking aids was allowed three weeks postoperatively, and the active hip flexion and abduction started about four weeks postoperatively. Ambulation with walking aids was allowed eight weeks postoperatively. Thereafter, patients were encouraged to walk without crutches twelve weeks postoperatively.

**Fig. 3:**
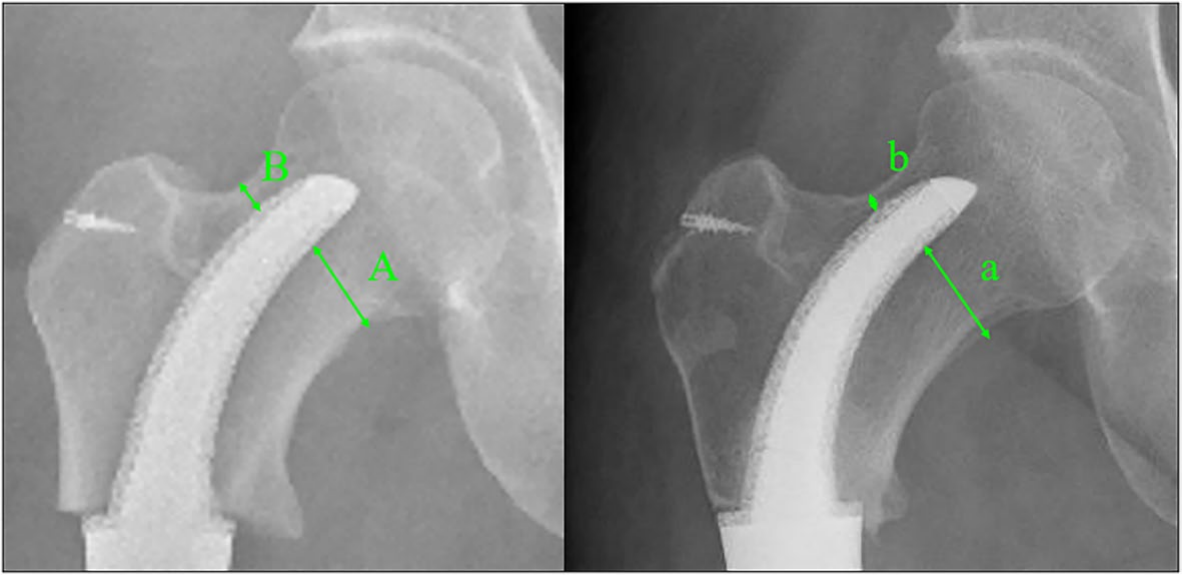
The prosthesis migration evaluation: the stem tip migration was calculated as [(a-A) + (B—b)]/2

All patients were followed up monthly for the first three months, then every three months for the first two years, then yearly. Follow-up items included: ① survival status, local recurrence, and distant metastasis; ② lower limb function (by using the Musculoskeletal Tumor Society score, MSTS); ③ pain relief (by using the visual analog scale, VAS); ④ postoperative complications (Infection, delayed wound healing, aseptic loosening, prosthesis breakage, periprosthetic fracture, etc.); ⑤ osteointegration of the bone/prosthesis interface (by using Tomosynthesis Shimadzu Metal Artefact Reduction Technology, T-SMART); and ⑥ prosthetic stem tip displacement. The extent of stem tip migration was evaluated by comparing the anteroposterior radiographs performed at different postoperative periods. As shown in Fig. 3, the stem tip displacement was calculated as [(a-A) + (B—b)]/2. A (B) represents the furthest distance between the inferior (superior) margin of the stem and the cortical margin of the femoral neck in the pelvis radiograph, taken two days after surgery; a (b) represents the distance measured at the same position in the pelvis radiograph, taken at the last follow-up.

## Results

### The FEM results

#### The stress and displacement of the normal femur

**Fig. 4:**
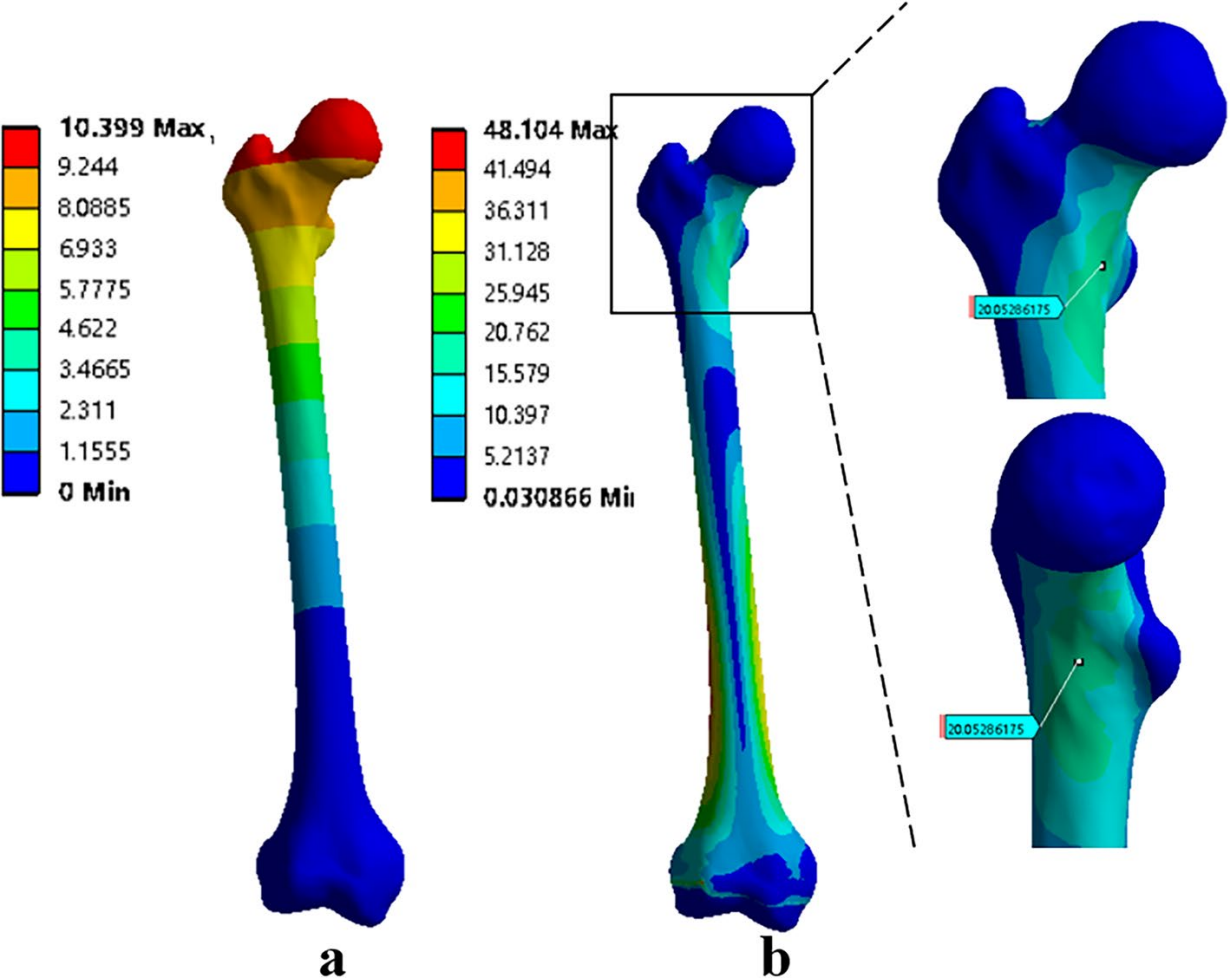
The displacement and stress distribution of the normal femur: **a** Displacement distribution **b** Stress distribution. The stress distribution of the femoral neck area was highlighted in the plots by black box, and the peak stress was 20.05 Mpa

As shown in Fig. 4b, the hip joint force applied to the femoral head was transmitted to the neck and from there to the shaft and the distal condyles. The stress distributed uniformly on the femur without evident stress concentration. The relatively high stress concentration was located at the inferior region of the femoral neck (20.05 Mpa) and the distal third of the femur shaft (48.1 Mpa). Moreover, the concentration region of maximum displacement located at the top of the femoral head (10.40 mm), and the displacement values gradually decreased from the proximal to the distal part of the femur (Fig. 4a). The results were similar to that of comparable studies [24, 25], which demonstrated the reliability of the finite element model.

#### The stress and displacement of short proximal femur segments after intercalary reconstruction

The maximum Von Mises stress was observed at the inferior region of the femoral neck in all the models. However, different levels of stress concentration were found between the four different models. The maximum Von Mises stress was 37.8 Mpa for Model# A, 13.57 Mpa for Model# B, 17.10 Mpa for Model# C, and 75.70 Mpa for Model# D, respectively. Additionally, there were different displacement levels of the remaining femur between different models. The peak displacement was 5.27 mm for Model# A, 6.48 mm for Model# B, 5.90 mm for Model# C, and 7.63 mm for Model# D respectively.

**Fig. 5:**
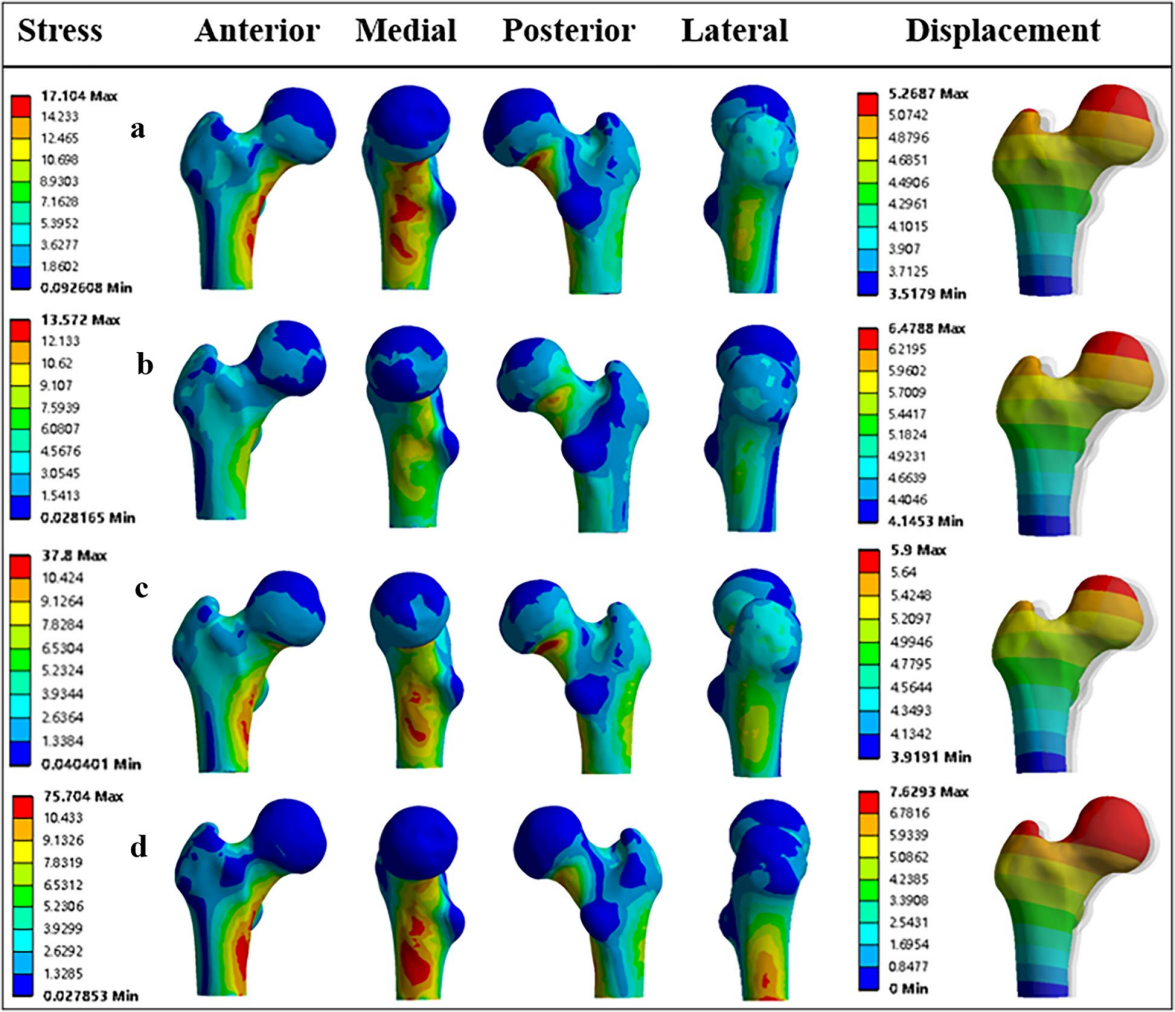
The displacement and stress distribution of the remaining femurs: **a** The stress was equally distributed, and no visible signs of stress concentration occurred in the remaining femur in Model#1 (by stem A). **b** Stress shielding can be observed at the femoral neck area in the remaining femur in Model#2 (by stem B). **c** Significant stress concentration can be observed in the remaining femur in model#3 (by stem C). **d** Remarkable stress concentration and high displacement can be both observed in the remaining femur in model#4 (by stem D)

For Model# A, by the reconstruction of segmental bone defects, the transmission mode of the femoral mechanics has been partly restored. Normal-like stress and displacement distribution patterns were observed in the remaining proximal femur segment. For Model# B, even though the overall stress and displacement distribution showed a similar trend as in the normal femur, noticeable stress shielding was observed near the femoral neck region, with Von Mises stress ranging from 6.08 Mpa to 13.57 Mpa. For Model# C, similar distribution patterns of stress and displacement were also observed. However, without significant stress shielding effect was found. For Model# D, the extent of stress concentration at the inferior region of the femoral neck was significantly higher, compared to the other three models. Moreover, Model# D has the highest displacement value of 7.63 mm (Fig. 5a-d).

#### The stress and displacement of the implants

**Fig. 6:**
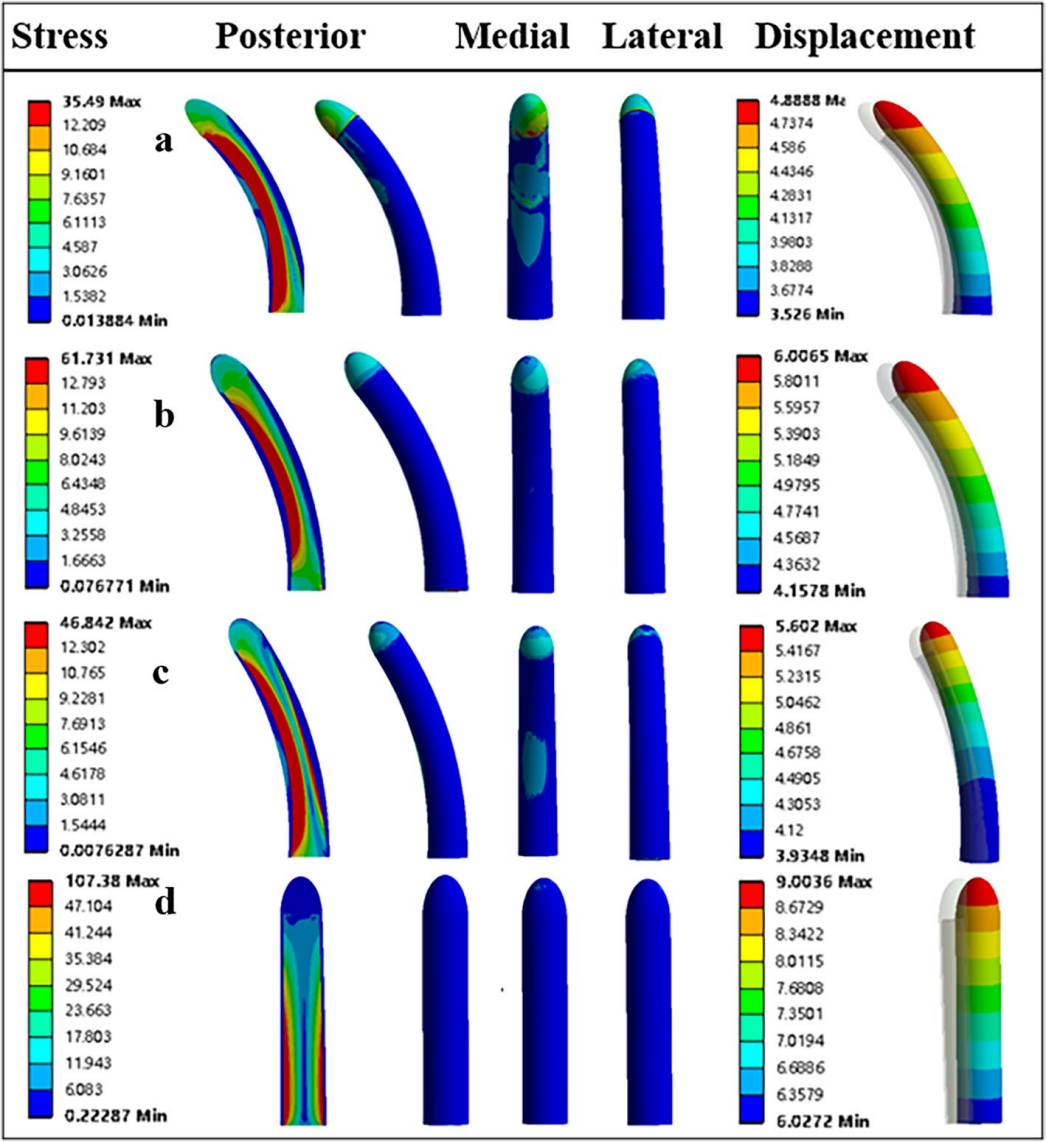
The displacement and stress distribution of the prosthetic stems: **a** The stress and displacement of stem A. **b** The stress and displacement of stem B. **c** The stress and displacement of stem C. **d** The stress and displacement of stem D

In all intercalary femoral replacement models, the stems have shown a similar overall trend in stress distribution. This trend can be very well captured by the cross-sectional image, as shown in Fig. 6: ① The inner part of the stem made of solid titanium shared the main stress of the whole loading, compared with the outer part of the stem made of porous titanium. ② Except for the femur reconstructed by the straight stem, all other curved stems showed an asymmetrical stress distribution; the stress concentration is more marked at the medial region of the stem, compared with the lateral region.

However, a different extent of stress concentration has been found on the stems of different intercalary femoral replacement models. The maximum Von Mises stress of the stem was 67.73 Mpa for Model# A, 35.49 Mpa for Model# B, 46.84 Mpa for Model# C, and 107.38 Mpa for Model# D, respectively. In addition, the peak displacement of the implants was 4.89 mm for Model# A, 6.01 mm for Model# B, 5.60 mm for Model# C, and 9.00 mm for Model# D, respectively (Fig. 6a-d).

### Clinical outcome

**Fig. 7:**
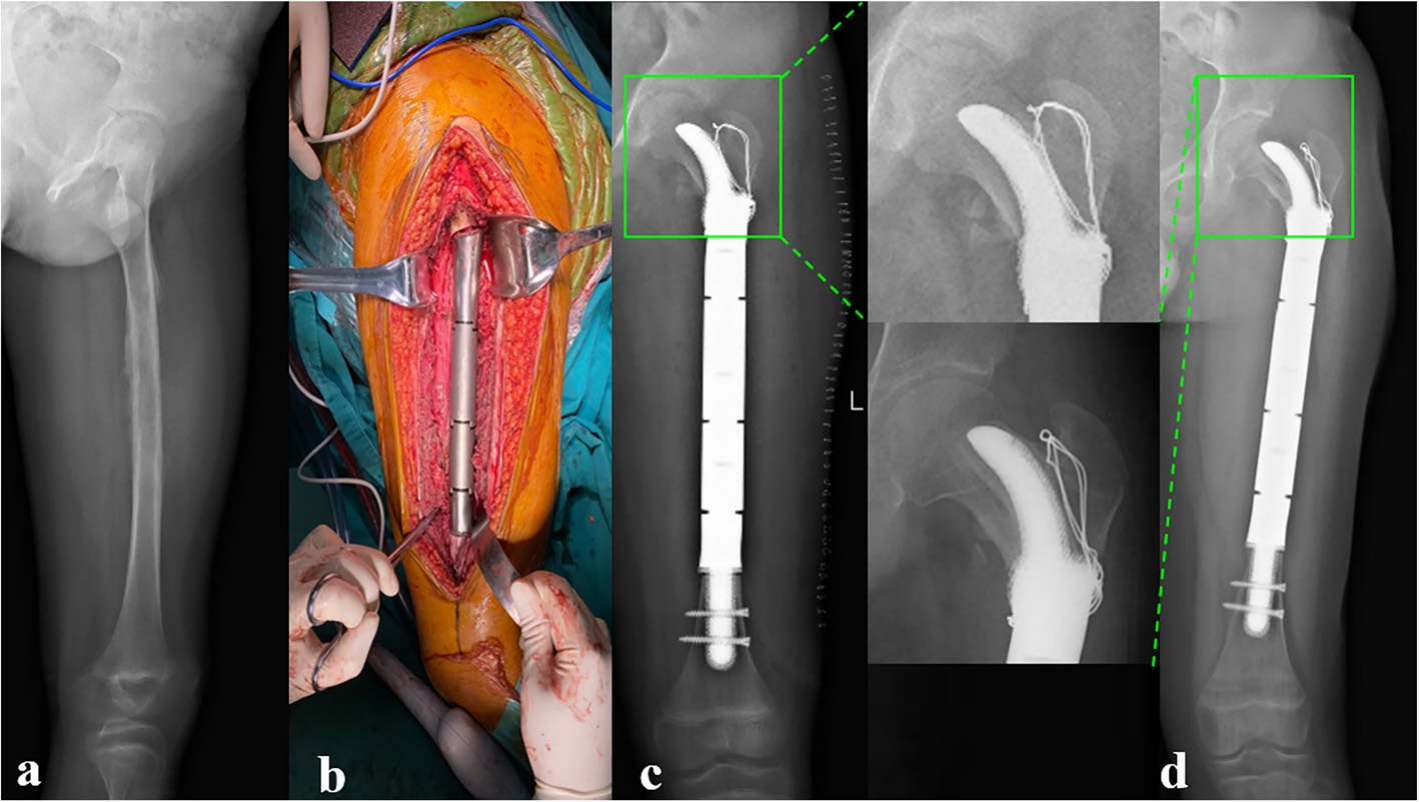
Preoperative and postoperative X-ray evaluations for typical case reconstructed by Stem B: **a** Preoperative X-ray of femur of Patient 2. **b** Intercalary femoral replacement was performed. **c** X-ray of femur taken at 2 days after surgery. **d** X-ray taken 24 months after surgery. The Stem tip migration occurred (green box)

All patients were followed up for a mean of 32.33 ± 9.12 months (range, 18–48). At the date of the last follow-up, all patients survived without tumor recurrence or metastasis. The mean MSTS (25.5 ± 1.3 vs. 18.67 ± 0.9) and mean VAS (0.5 ± 0.9 vs. 5.4 ± 1.0) scores were significantly improved, compared with preoperative records. No complications occurred during the follow-up period except for aseptic loosening of stem B in one patient (Fig. 7a-d); however, no revision surgery was performed due to the function being acceptable.

**Fig. 8:**
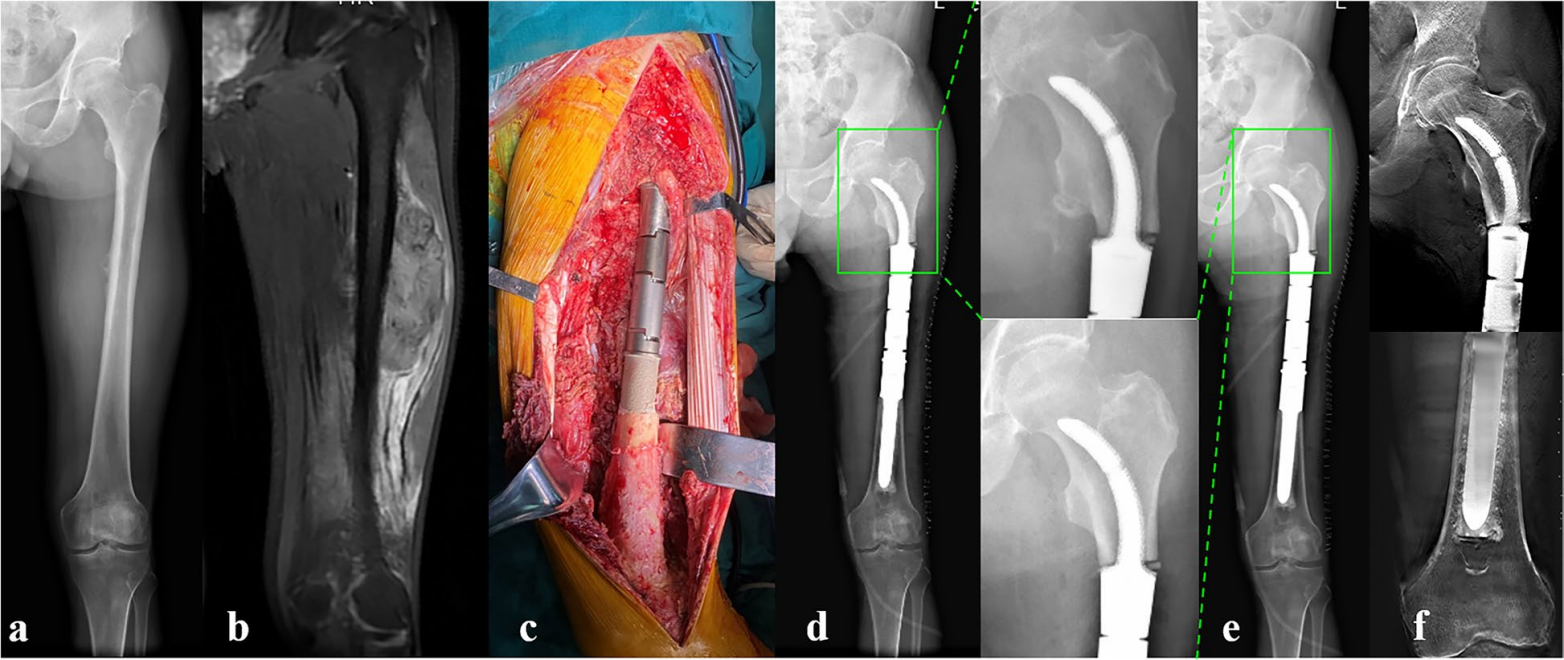
Preoperative and postoperative X-ray evaluations for typical case reconstructed by Stem A: **a** Preoperative X-ray of femur of Patient 7. **b** Preoperative MRI of the femur. **c** Intercalary femoral replacement was performed. **d** X-ray taken 1 day after surgery. **e** X-ray taken 12 months after surgery. Note the stem tip placement without any drift (green box). **f** T-SMART taken at 12 months after surgery, and the osseointegration of the implant-bone interface can be observed

**Fig. 9:**
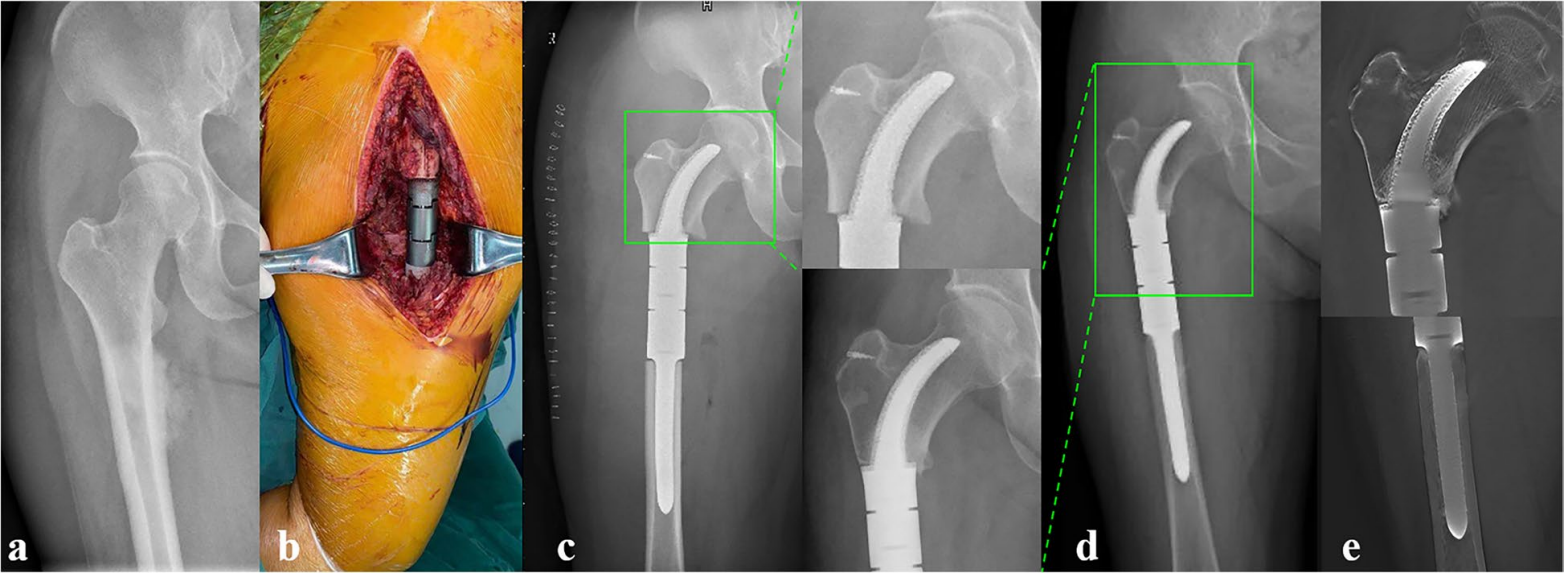
Preoperative and postoperative X-ray evaluations for typical case reconstructed by Stem C: **a** Preoperative X-ray of femur of Patient 4. **b** Intercalary femoral replacement was performed. **c** X-ray taken 1 day after surgery. **d** X-ray taken 12 months after surgery. No obvious stem tip migration occurred (green box). **e** T-SMART taken at 12 months after surgery, and the osseointegration of the implant-bone interface can be observed

With respect to radiographic outcome, all rhino horn-designed cementless stems except the one with aseptic loosening showed good radiologic osseointegration without fretting wear or radiolucent line. However, there were differences found in the stem tip displacement according to stem type. The mean value of stem tip displacement of Stem A was 0.63 ± 0.8 mm, of Stem C was 1.0 ± 0.8 mm, and of Stem B was 2.0 ± 1.4 mm, which was the highest. Detailed results of patient follow-up are summarized in Table 4, and three typical cases reconstructed by Stem A, B, and C are shown in Fig. 8(a-f), Fig. 7(a-d), and Fig. 9(a-e), respectively.

**Table 4:** Clinical follow-up data of patients

Patients	VAS score		MSTS score		Recurrence or metastasis	Postoperative complications	Prosthetic stem tip migration (mm)
	Preop	Last follow-up	Preop	Last follow-up			
1	5	0	18	26	None	None	2.0
2	5	0	19	25	None	None	4.0
3	4	0	19	27	None	None	1.0
4	6	0	19	25	None	None	2.0
5	7	0	18	24	None	None	1.0
6	7	0	20	26	None	None	1.0
7	4	2	19	25	None	None	1.5
8	5	2	18	24	None	None	0.0
9	6	0	19	24	None	None	0.0
10	6	0	20	25	None	None	1.0
11	5	2	17	28	None	None	1.0
12	5	0	18	27	None	None	0.0

## Discussion

For patients with SSPF following extensive femoral diaphyseal tumor resection, the intercalary prosthesis reconstruction may seem to be an alternative. At present, the most commercially available intercalary prosthesis provided a straight femoral stem design. Meanwhile, considering the insufficient length of the remaining bone of the SSPF, only a short femoral stem could be inserted into the SSPF. In such a scenario, cementing a short straight stem of a modular intercalary endoprosthesis into the SSPF for fixation would be expected to have a high rate of aseptic loosening and implant failure [8]. Clinical studies have verified this, with poor results in lower extremity endoprosthetic reconstruction when dealing with tumorous massive defects [8, 20]. Coherently, FEMs presented in the present study showed that the remaining femur reconstructed by the straight stem had the highest extent of stress concentration and displacement (Fig. 5d), which indicated poor stability of the surgical reconstruction and an increased possibility of mechanical failure [24, 25].

In our study, a rhino horn-designed uncemented stem has been devised to overcome the limitation of the standard modular intercalary endoprosthesis. This newly designed stem was customized according to the preoperative imaging data. Thus, it could match well with the curvature of the SSPF and provide a more physiological load transmission in the femoral stem. As the FEA results showed, normal-like stress distribution patterns were observed in the femurs reconstructed by the rhino horn-shaped stems (Fig. 5a-c). Moreover, the rhino horn-shaped design can provide enough primary stability by increasing the host bone-stem interface and decreasing the TLP/SL ratio, and it can generate strong derotational forces due to the space constraints while the effect is however absent for the standard straight stem [26]. Specifically, we found that the deformation and maximal displacement in model A are the lowest, while model B has the highest displacement level among the three curved stem models. This FEA result is consistent with our clinical and X-ray findings. In the clinical study, loosening of the stem occurred in one patient with Stem B prosthesis. Moreover, there was a general trend toward a higher displacement in the patients with Stem B prostheses; they had the highest mean value of stem tip displacement compared to other patients with Stem A or Stem C prostheses. With respect to the stress distribution of the implant, Model #A performed the best among all the models overall. There was neither significant stress shielding nor stress concentration found in the remaining femur reconstructed by Stem A, as has been found in other types of stems. A possible explanation for these results might be that the position of Stem A was closer to the inferior region of the femoral neck, which has a strong trabecular architecture and thicker cortices to resist tensile or shearing forces applied to the neck through the head [27, 28]. Inserting the stem into this area with a denser structure makes the fixation system more stable and more adaptive to the physiological stress transmission.

The rhino horn-designed uncemented stem has another remarkable advantage: low risk of aseptic loosening. There are various surgical methods described in the literature that would be available for the reconstruction of the SSPF, including the custom-made/modular megaprosthesis, APC remonstration, and CPS implant. Most of them usually suffer from high rates of aseptic loosening. George et al. [13] retrospectively reviewed 50 cases using compressive osseointegration fixation of endoprostheses for SSPF remaining after extensive tumor resection. Seven (14%) were the result of aseptic failure during a median follow-up of 66 months. In another 7-year follow-up study, Stevenson et al. [7] reported that three of ten (30%) patients who underwent endoprosthetic reconstruction using a short medullary stem with extra-cortical plate suffered aseptic loosening. With respect to previous works, only one case of aseptic loosening of the femoral stem was observed in our study. Encouraging results were registered also for the radiographic outcome. All rhino horn-designed uncemented stems showed excellent osseointegration. Most studies agreed that insufficient osseointegration is the major cause of orthopaedic implant loosening [29–31]. Therefore, it is reasonable to assume that the likelihood of late loosening would be low when bone ingrowth into the porous coating of the stem has taken place [32]. In addition, our low rate of loosening may also be related to the stem fixation type. The mode of fixation, cemented vs uncemented, is of great importance. Most studies have shown that cemented stems have a greater risk of aseptic loosening, while the uncemented stems may be advantageous due to bone in-growth surface. Thus, we utilized an uncemented short stem design to decrease the possibility of prosthesis loosening [33–35].

In general, the consistency between the finite element results and the follow-up study results demonstrated that the rhino horn-designed stem having a suitable stem tip position is important for the stress transmission after reconstruction. Inserting the curved stem into the femoral neck region where the stem tip is slightly above or below the femoral head center would be expected with a lower mechanical failure risk.

## The limitation and expectation

Limitations exist with our study. First, the major shortcomings of this study are the relatively short follow-up time and the small number of patients who were enrolled from a single hospital. For this reason, we were unable to conduct further stratified analyses of some variables such as the osteotomy length, and the proximal femur length after tumor resection. A longer follow-up time and more cases are required for verifying the long-term outcome; a future multicentre study is needed. Second, in the finite element study, the nonhomogeneous, inelastic, and nonlinear material of the bone and the implants were overlooked, requiring further research and consideration. Finally, the aseptic loosening is the result of a combination of various factors. The patient with aseptic loosening was younger and had thinner cortical bone and a large-segment bone defect after tumor resection. These are possible reasons causing aseptic loosening. However, patients in other groups with similar demographic characteristics did not develop similar complications. It may, to some degree, confirm the reliability of our conclusions.

## Conclusion

Thanks to the matchable shape and osteointegration ability, the rhino horn-shaped uncemented stem is associated with a low complication rate and good lower extremity functions. Moreover, Inserting the rhino horn-shaped stem into the femoral neck region where the stem tip is slightly above or below the femoral head center would be a better strategy to prevent mechanical failure. Therefore, the customized intercalary prosthesis with the rhino horn-designed uncemented stem might be a reasonable alternative for the reconstruction of ultrashort proximal femur segments following extensive tumor resection.

## Data Availability

The datasets used and analyzed during the current study are available from the corresponding author on reasonable request.

## Abbreviations

SSPF: Short bone-segments in proximal femur
FEM: Finite Element Method
APC: Allograft Prosthetic Composite
CPS: Compress® Compliant Pre-stress implant
ROC: Radius of Curvature
MSTS: Musculoskeletal Tumor Society Score
VAS: Visual Analog Scale
T-SMART: Tomosynthesis Shimadzu Metal Artefact Reduction Technology
